# Development, characterization and use of microsatellite markers for genetic analysis of Cashew tree (*Anacardium occidentale*)

**DOI:** 10.1186/1753-6561-5-S7-P157

**Published:** 2011-09-13

**Authors:** Gláucia Buso, Natalia Lamas, José Jaime Cavalcanti, Marco Antonio Ferreira

**Affiliations:** 1 Embrapa Cenargen, Brazilia, DF, Brazil; 2 Embrapa Agroindustria Tropical, Fortaleza, CE, Brazil

## Background

The cashew tree (*Anacardium occidentale*) is found throughout the Brazilian territory, although it is better adapted to the northeastern coast climate. This fruit crop is essential to the agroindustry of the States of Ceará, Piauí and Rio Grande do Norte, where around 95% of the production is harvested and where the whole processing of the chestnut is done. It currently represents 157 million dollars in exports of nuts. Despite its socio-economic importance it still lags behind in the adoption of breeding technologies. There is very little information about the genetics of this species and no molecular tools yet developed. In this work we developed and characterized a set of microsatellite markers for *Anacardium occidentale* from the construction of genomic libraries enriched for repetitive sequences.

## Material and methods

The markers developed were used in studies of genetic relationships among samples of the germplasm bank, and saturation of existing genetic maps of the species. Genomic libraries enriched for microsatellites were constructed and out of the 5,472 selected clones, 540 were sequenced and analyzed for the presence of microsatellites. A total of 117 sequences containing microsatellites were selected to design PCR primers and screen the resulting markers. One hundred pairs of primers were synthesized and initially tested for amplification in agarose gel. Fourteen markers were selected and characterized by fluorescent detection in an ABI 377 sequencer based on genotyping a representative set of 35 samples derived from the germplasm collection. Genetic parameters such as observed heterozygosity (Ho), expected heterozygosity (He), polymorphic information content (PIC) and power of exclusion (PE) for paretage testing were estimated.

## Results

Great variability in analytical performance was seen among the tested microsatellite markers.Marker AOBR48, for example, proved to be the marker with the highest polymorphic information content. Analysis of accessions from the germplasm collection using microsatellite markers indicated that cashew and a commonly used dwarf clone likely have distinct origins, since they were separated into different groups based on similarity coefficients. Additionally, A. microcarpum, used as an outgroup control, clustered with accessions of the giant type. An F1 population derived from a cross between the dwarf cashew variety (CCP1001) and the giant type (CP 96) was used to map these microsatellites. Among the hundred new markers developed in this study, 11 showed segregation of alleles from the female parent (CCP 1001) in the F1 population, while 21 showed segregation of alleles from the male parent (CP 96). Only three of the 100 tested markers were fully informative showing segregation of alleles from both parents in the F1 population. In total 29 markers could be mapped in this population and 11 markers were positioned in both, the male and female maps.

## Conclusions

An initial set of microsatellites was developed for the Cashew tree. These new markers can be useful for a number of applications in germplasm analysis and breeding. The identification of duplicate accessions in the current germplasm collection, the study of genetic diversity in natural populations, represent immediate potential applications to better understand the existing diversity available to breed better clones. Genetic mapping of QTLs for some economically important traits such as increased yield and quality of fruits and cashew, and resistance to diseases could be future targets provided a higher marker density is achieved.

